# The role of *APOE* in cognitive trajectories and motor decline in Parkinson’s disease

**DOI:** 10.1038/s41598-021-86483-w

**Published:** 2021-04-09

**Authors:** Sungyang Jo, Seon-Ok Kim, Kye Won Park, Seung Hyun Lee, Yun Su Hwang, Sun Ju Chung

**Affiliations:** 1 Department of Neurology, Asan Medical Center, University of Ulsan College of Medicine, 88, Olympic-ro 43-gil, Songpa-gu, Seoul, 05505 Korea; 2 Department of Clinical Epidemiology and Biostatistics, Asan Medical Center, University of Ulsan College of Medicine, 88, Olympic-ro 43-gil, Songpa-gu, Seoul, Korea

**Keywords:** Diseases, Neurology

## Abstract

We aimed to investigate the role of the *APOE* genotype in cognitive and motor trajectories in Parkinson’s disease (PD). Using PD registry data, we retrospectively investigated a total of 253 patients with PD who underwent the Mini-Mental State Exam (MMSE) two or more times at least 5 years apart, were aged over 40 years, and free of dementia at the time of enrollment. We performed group-based trajectory modeling to identify patterns of cognitive change using the MMSE. Kaplan–Meier survival analysis was used to investigate the role of the *APOE* genotype in cognitive and motor progression. Trajectory analysis divided patients into four groups: early fast decline, fast decline, gradual decline, and stable groups with annual MMSE scores decline of − 2.8, − 1.8, − 0.6, and − 0.1 points per year, respectively. The frequency of *APOE* ε4 was higher in patients in the early fast decline and fast decline groups (50.0%) than those in the stable group (20.1%) (*p* = 0.007). *APOE* ε4, in addition to older age at onset, depressive mood, and higher H&Y stage, was associated with the cognitive decline rate, but no *APOE* genotype was associated with motor progression. *APOE* genotype could be used to predict the cognitive trajectory in PD.

## Introduction

Cognitive impairment in Parkinson’s disease (PD) is important, because it negatively affects patients’ quality of life, and increases caregiver burden^1^. Patients with PD show a heterogeneous cognitive decline in terms of decline rate, and the time interval from cognitive deficit and PD diagnosis^2,3^. Variations in the patterns of cognitive decline in PD are important in predicting the prognosis and might be considered when designing clinical trials^4^. Longitudinal studies are required to observe the divergent trajectory of cognition in patients with PD. However, most of the studies that investigated the cognition of patients with PD were cross-sectional studies^5^, which were unable to discriminate between patients with a fast decline in cognition and those with a slow decline.

Group-based trajectory modeling has been used to summarize the variation pattern and to identify prognostic factors for chronic disease including dementia^6,7^, hypertension^8^, diabetes mellitus^9^, chronic kidney disease^10^, and juvenile idiopathic arthritis^11^. Group-based trajectory modeling involves data-driven analysis, rather than using arbitrarily selected thresholds^12^. Investigation of cognitive trajectories using trajectory modeling was rarely performed in PD. Revealing prognostic factors for cognitive trajectories could enable the prediction of disease progression.

Several risk factors including environmental and genetic factors were identified in previous studies. Environmental factors include cerebrovascular disease, diabetes mellitus, obesity, cardiac disease, alcohol consumption, and smoking^13^. Genetic risk factors include *GBA* mutation, while common variants of Apolipoprotein E (*APOE), MAPT, COMT* and *SNCA* showed conflicting results^14^. *APOE* ε4 is a well-established risk factor for sporadic Alzheimer’s disease^15^. Recent preclinical data suggest that *APOE* ε4 regulates α-synuclein pathology and related toxicity, which is the pathogenic hallmark of PD^16,17^. Current evidence regarding the role of *APOE* in cognitive and motor decline in PD is unclear; some studies have identified *APOE* ε4 as a risk factor for dementia in PD^18–20^, while other studies found no association^21,22^. Although *APOE* ε4 was not associated with motor severity features of PD in clinical studies^18,19^, mice with *APOE* ε4 allele showed poor motor performance^17^, while mice with *APOE* ε2 allele showed increased survival and motor performance^16^. Moreover, as most of the previous studies on *APOE* were cross-sectional studies, the relationship between *APOE* and cognitive trajectories in PD remains unexplained. Considering that novel emerging therapeutic strategies to target *APOE* have been introduced to prevent *APOE* ε4-induced tau and amyloid-beta pathology^15^, the role of the *APOE* genotype in the progression of cognitive and motor symptoms in PD should be investigated.

In this study, we aimed to investigate longitudinal cognitive trajectories in patients with PD over a median follow-up period of 7 years using group-based trajectory modeling and to evaluate the role of the *APOE* genotype in the progression of cognitive and motor symptoms in PD.

## Results

### Cognitive trajectories of Parkinson’s disease

**Figure 1:**
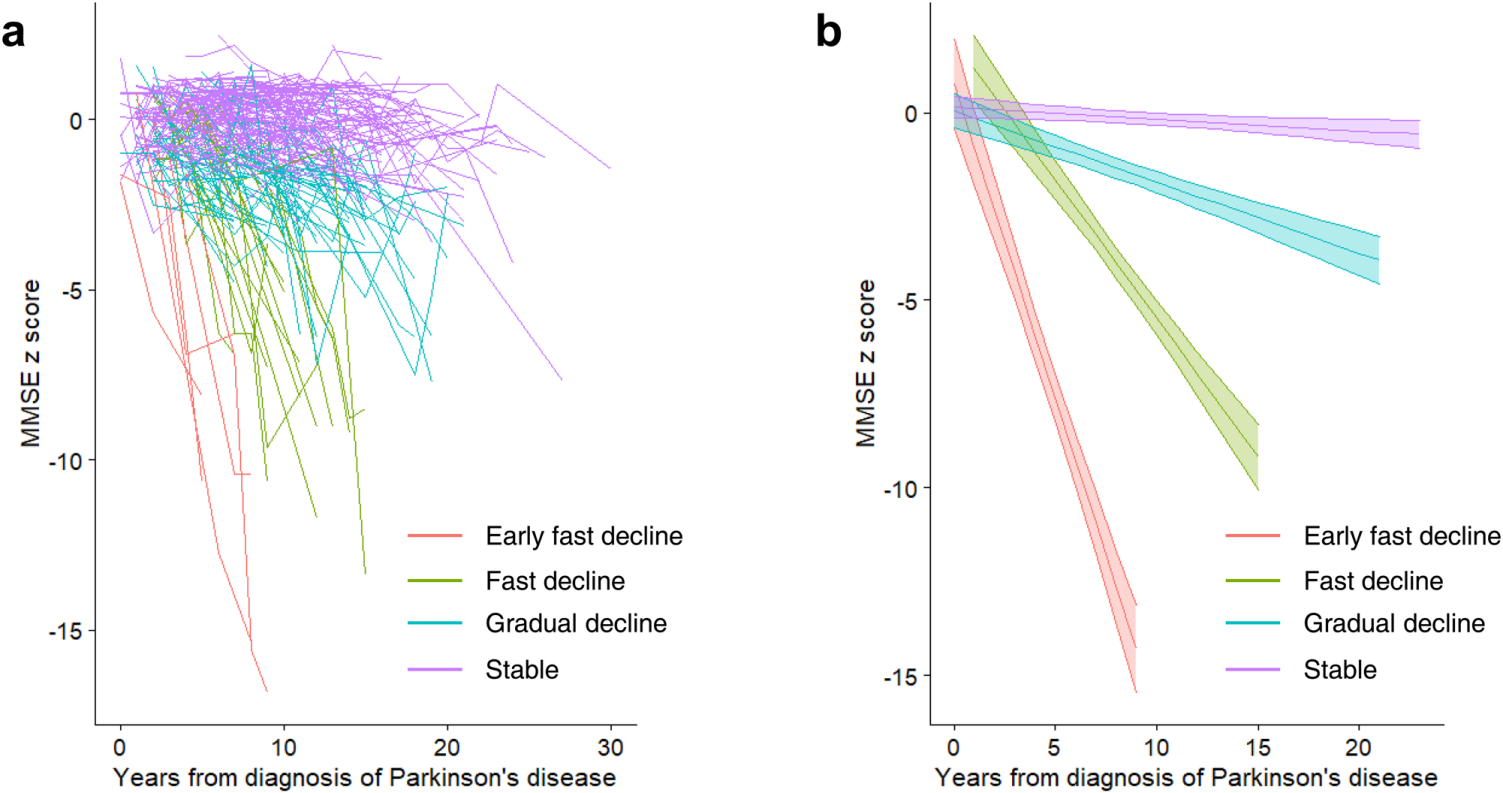
Cognitive trajectories in patients with Parkinson’s disease. (**a**) Spaghetti plot of individual MMSE scores, (**b**) Cognitive trajectories are shown with 95% confidence intervals (shaded area). MMSE = Mini-Mental State Examination.

The mean age at onset (SD) of the study population was 57.4 (9.6) years, and 130 (51.4%) of patients were female. The median follow-up duration was 7 years (IQR 6.0–9.0). The spaghetti plot of longitudinal Mini-Mental State Exam (MMSE) scores of the individual study population revealed a clinical heterogeneity of cognitive decline in PD (Fig. 1a). According to the Bayesian information criterion (BIC) values of the group-based trajectory modeling, the four-group model was found to be the best (Fig. 1b). Among 253 patients, 6 patients (2.4%), 16 patients (6.3%), 62 patients (24.5%), and 169 patients (66.8%) were classified as “early fast decline”, “fast decline”, “gradual decline”, and “stable” groups, respectively. (Table 1). Motor trajectories are depicted in Supplementary Table S1.

**Table 1:** Patient demographics and clinical characteristics at study enrollment.

	Early fast decline (n = 6)	Fast decline (n = 16)	Gradual decline (n = 62)	Stable (n = 169)	*p*
Age at disease onset, mean ± SD	65.7 ± 7.9	60.6 ± 9.8	61.1 ± 9.1	55.5 ± 9.3†	0.002
Female, n (%)	1 (16.7%)	7 (43.8%)	30 (48.4%)	92 (54.4%)	0.25
Education (years), median (IQR)	16.0 (12.0–16.0)	12.0 (9.0–16.0)	9.0 (6.0–13.0)	9.0 (6.0–12.0)‡	0.009
Disease duration at enrollment (y), median (IQR)	2.0 (0.0–3.0)	1.5 (1.0–5.0)	4.0 (0.0–6.0)	3.0 (1.0–7.0)	0.46
Initial MMSE (z), median (IQR)	− 1.4 (− 1.6 to 0.1)	− 0.7 (− 1.6 to 0.3)	− 0.9 (− 1.4 to 0.0)	− 0.1 (− 0.7 to 0.5)§	< 0.001
**Vascular risk factor at study enrollment, n (%)**					
Hypertension	3 (50.0)	3 (18.8)	21 (33.9)	36 (21.3)	0.10
Diabetes mellitus	1 (16.7)	2 (12.5)	9 (14.5)	15 (8.9)	0.61
Hyperlipidemia	0 (0.0)	2 (12.5)	6 (9.7)	26 (15.4)	0.52
Cardiac disease	1 (16.7)	1 (6.2)	6 (9.7)	8 (4.7)	0.39
***APOE*** **genotype, n (%)**					
ε4 carrier	4 (66.7)	7 (43.8)	13 (21.7)	34 (20.1)	0.011¶
ε2 carrier	0 (0.0)	3 (18.8)	5 (8.3)	18 (10.7)	0.53
**Environmental risk factor, n (%)**					
Heavy alcohol drinking	0 (0.0)	1 (6.2)	3 (4.8)	11 (6.5)	0.97
Smoking	2 (33.3)	2 (12.5)	10 (16.1)	38 (22.5)	0.63
Use of pesticide	0 (0.0)	2 (12.5)	4 (6.5)	20 (11.8)	0.53
**Nonmotor symptom, n (%)**					
Depressive mood	5 (83.3)	8 (50.0)	31 (50.0)	75 (44.4)	0.269
Pain	4 (66.7)	6 (37.5)	31 (50.0)	73 (43.2)	0.503
Fatigue	5 (83.3)	10 (62.5)	37 (59.7)	86 (50.9)	0.263
Psychosis	2 (33.3)	1 (6.2)	10 (16.1)	19 (11.2)	0.273
Autonomic dysfunction	5 (83.3)	10 (62.5)	36 (58.1)	82 (48.5)	0.189
REM sleep behavior disorder	4 (66.7)	7 (43.8)	43 (69.4)	108 (63.9)	0.303
H&Y stage, median (IQR)	2.0 (2.0–3.0)	2.0 (2.0–2.5)	2.0 (2.0–2.5)	2.0 (2.0–2.0)	0.119
Levodopa equivalent dose	675.0 (420.0–925.0)	650.0 (425.0–805.0)	707.5 (550.0–884.0)	700.0 (450.0–940.0)	0.730

*H&Y* Hoehn and Yahr; *MMSE* mini-mental state exam; *RBD* Rapid eye movement sleep behavior disorder.

^†^Significant difference compared with the early fast decline and gradual decline groups, using Tukey’s post-hoc test.

^‡^Significant difference compared with the fast decline group using Tukey’s post-hoc test.

^§^Significant difference compared with the gradual decline group using Tukey’s post-hoc test.

^¶^No significant difference in post-hoc analysis after Bonferroni correction.

The mean age at onset of patients in the stable group (55.5 ± 9.3) was significantly lower than those in the early fast decline (65.7 ± 7.9) and gradual decline groups (61.1 ± 9.1) (*p* = 0.002) (Table 1). There were no differences in sex and disease duration. Education level was significantly higher in the fast decline group (median 12.0, C.I 9.0–16.0) than in the stable group (median 9.0, C.I 6.0–12.0) (*p* = 0.009). There were no differences in the frequency of vascular risk factors, heavy alcohol drinking, smoking, and pesticide use between the four groups. There were more patients with the *APOE* ε4 allele in the early fast decline and fast decline groups than in the stable group (*p* = 0.011), but no statistically significant difference was identified in Tukey’s post-hoc analysis. However, the frequency of *APOE* ε4 in the combined group of early fast decline and fast decline groups (50.0%) was statistically higher than in the stable group (20.1%) using Tukey’s post-hoc analysis (*p* = 0.007). The frequency of *APOE* ε2 was not significantly different between the four groups (*p* = 0.534). Non-motor symptoms, initial Hoehn and Yahr (H&Y) stage, and levodopa equivalent dose were not significantly different between the four groups. Homozygous *APOE* ε4 allele was found in only two patients (one in the early fast decline, and one in gradual decline groups). There was no patient with homozygous *APOE* ε2 allele.

### Prognosis of cognitive trajectories

Among 253 patients in the study population, 61 patients (24.1%) developed dementia during a median follow-up period of 7 years (IQR 6.0–9.0 years) (Supplementary Table S2). The conversion rate was significantly lower in the stable group compared to the remaining groups (*p* < 0.001). The median annual change in MMSE raw score in the whole study population was − 0.2 points per year, and − 2.8, − 1.8, − 0.6, and − 0.1 points per year for the early fast decline, fast decline, gradual decline, and stable groups, respectively.

### Factors associated with the annual change in MMSE

The univariate linear regression analysis to identify factors associated with the annual change in MMSE score is depicted in Supplementary Table S3. The multivariate linear regression analysis indicated that age at disease onset (*p* < 0.001), *APOE* ε4 carrier status (*p* < 0.001), the depressive mood at the time of study enrollment (*p* = 0.004), and H&Y stage at study enrollment (*p* < 0.001) were negatively associated with annual changes in MMSE z-score (Table 2).

**Table 2:** Stepwise linear regression showing factors significantly associated with the mean annual change in MMSE in patients with Parkinson’s disease.

	B	SE	*p*
Age at disease onset, years	− 0.030	0.005	< 0.001
*APOE* ε4 carrier	− 0.470	0.103	< 0.001
Depressive mood	− 0.258	0.088	0.004
H&Y score at study enrollment	− 0.301	0.080	< 0.001

*H&Y* Hoehn and Yahr; *MMSE* mini-mental state exam.

### The role of the *APOE* ε4 allele in the annual cognitive and motor decline

**Figure 2:**
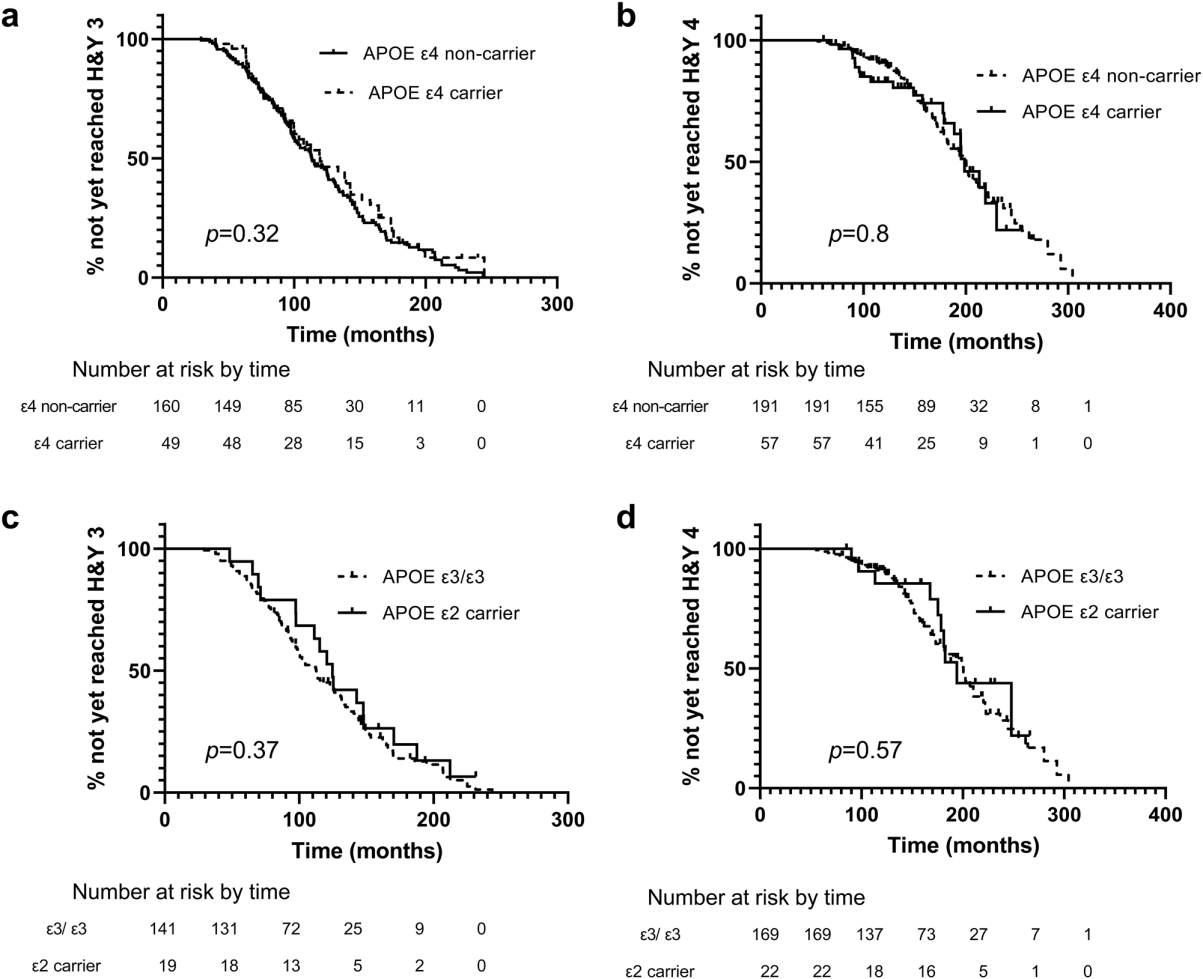
Motor progression according to the *APOE* genotype. (**a**) Kaplan–Meier curves for motor progression from diagnosis to the onset of H&Y stage 3 between *APOE* ε4 carriers and non-carriers. (**b**) Kaplan–Meier curves for motor progression from diagnosis to the onset of H&Y stage 4 between *APOE* ε4 carriers and non-carriers. (**C**) Kaplan–Meier curves for motor progression from diagnosis to the onset of H&Y stage 3 between *APOE* ε2 carrier and ε3/ε3 carriers. (**D**) Kaplan–Meier curves for motor progression from diagnosis to the onset of H&Y stage 4 between *APOE* ε2 carriers and ε3/ε3 carriers.

**Figure 3:**
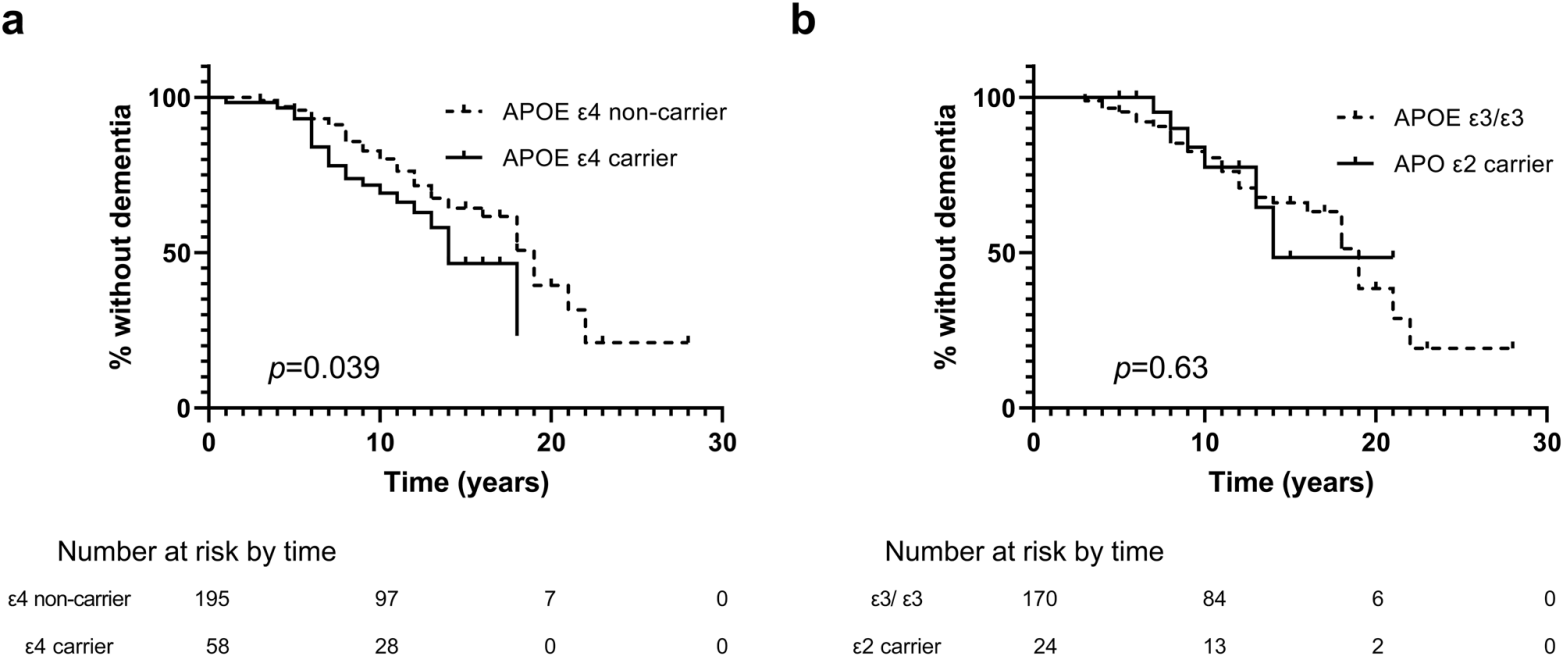
Progression to dementia according to the *APOE* genotype. (**a**) Kaplan–Meier curves from diagnosis to the onset of dementia between *APOE* ε4 carriers and non-carriers. (**b**) Kaplan–Meier curves from diagnosis to the onset of dementia between *APOE* ε2 carriers and ε3/ε3 carriers.

The log-rank test showed that motor progression from PD diagnosis to the onset of H&Y stage 3, and from PD diagnosis to the onset of H&Y stage 4 were not associated with the *APOE* ε4 (Fig. 2a,b) nor ε2 genotypes (Fig. 2c,d). *APOE* ε4 was associated with progression to dementia (*p* = 0.039) (Fig. 3a). *APOE* ε2 was not associated with progression to dementia (Fig. 3b).

### Correlation between the cognitive and motor decline

**Figure 4:**
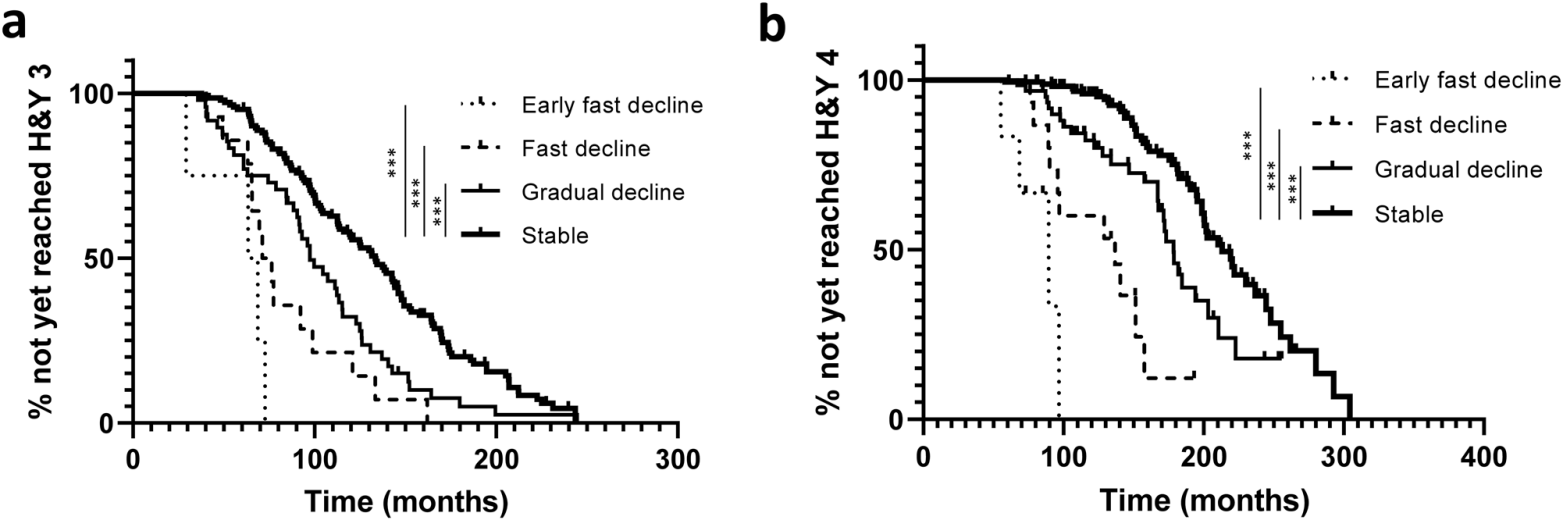
Progression of Hoehn and Yahr (H&Y) stage according to cognitive trajectories. (**a**) Kaplan–Meier curves for motor progression from diagnosis to the onset of H&Y stage 3 according to cognitive trajectories. (**b**) Kaplan–Meier curves for motor progression from diagnosis to the onset of H&Y stage 4 according to cognitive trajectories. ***p < 0.001.

The log-rank test showed that cognitive trajectories were significantly associated with motor progression from PD diagnosis to the onset of H&Y stage 3, and to the onset of H&Y stage 4 (*p* < 0.001) (Fig. 4). Compared with patients in the stable group, patients in early fast decline, fast decline, and gradual decline groups showed significantly faster motor progression (*p* < 0.001 in all analyses) (Fig. 4). The Cox proportional hazard model showed that patients in the early fast decline group had a hazard ratio of 8.25 (95% CI, 3.45–19.75; *p* < 0.001) in terms of motor progression to H&Y stage 3 compared with the stable group, after adjusting for age at onset and disease duration (Supplementary Table S4).

## Discussion

By using longitudinal follow-up data of patients with PD, we identified cognitive trajectories of PD and the role of the *APOE* genotype in the progression of cognitive and motor symptoms. Patients with PD were classified into four distinct cognitive trajectories, including early fast decline, fast decline, gradual decline, and stable groups, which had a strong positive correlation with the prognosis. Patients with PD who showed fast cognitive decline were older age at onset, and had a higher frequency of *APOE* ε4 when compared with patients who had stable cognition. However, neither *APOE* ε4 nor *APOE* ε2 was associated with motor decline progression.

In this study, we found a highly heterogeneous progression of cognitive symptoms, and grouped patients into four cognitive trajectories. Most of the previous longitudinal studies assessed conversion to dementia, rather than evaluating the rate of cognitive decline^23–25^. In the CamPaIGN study, 46% of patients with PD developed dementia during 10 years of follow-up, while 23% of patients showed good outcomes without dementia or postural instability^23^. Dichotomizing cognition into normal and dementia is less sensitive to cognitive progression, because a large variety of impairment severities is collapsed together. Using trajectory analysis, we observed divergent cognitive decline patterns across time. In addition, we found some prognostic factors for cognitive trajectories in PD. Patients who would show a fast cognitive decline (“early fast decline” and “fast decline” groups) were older age at PD onset, and had a higher frequency of *APOE ε4*, compared with PD patients with stable cognition. A high level of education in the fast cognitive decline group was unexpected. As education was not associated with an annual decrease in MMSE in multivariate analysis (Table 2), we can assume that a high level of education in the early fast decline group is caused by confounding factors or a small number of participants in the group. Also, we thought that patients in the fast cognitive decline group have more genetic or environmental risk factors such as *APOE* ε4, which might override the protective effect of education.

The frequency of the *APOE* ε4 allele in patients with fast cognitive decline was higher than in patients with stable cognition. Although *APOE* ε4 is a well-established risk factor for sporadic Alzheimer’s disease^15^, previous studies showed inconsistent results on its association with dementia in PD. Some studies have found that *APOE* ε4 is a risk factor for dementia in PD (PD-D)^18–20^, other studies have found no association with dementia in PD^22,26–28^. Inconsistent results might be caused by different measurements of cognitive decline, characteristics and number of the study population, and study design. Some negative studies had a small number of patients with PD-D (less than 30)^22,26–28^, while most positive studies had more than 50 patients with PD-D^18–20^. In two negative studies, age at PD onset of the study population was younger (in 50 s) than what we saw in the fast decline groups^29,30^. As the annual cognitive decline rate is slower in PD patients with younger age at onset, than those with older age at onset, younger patients who would decline to dementia later, might have been misclassified as normal cognition when they were enrolled in the early stage of the disease. This misclassification is inevitable in cross-sectional studies, which accounts for most previous studies. By using longitudinal data, we clarified that *APOE* ε4 contributes to fast cognitive decline in PD.

An experimental study showed that α-synuclein-overexpressing *APOE* ε*4* mice showed greater deficits in balance and motor coordination than α-synuclein-overexpressing *APOE* ε*3* mice^17^. However, we found that *APOE* ε4 allele was not associated with faster motor decline in this study. This is consistent with previous studies^18,19^. *APOE* was not associated with fast progression of motor symptom in PD in a genome-wide association study, consistent with our results^31^. We also found no evidence for a protective effect of *APOE* ε2 allele for either cognition or motor decline, unlike experimental studies that showed increased survival and improved motor performance in an *APOE* ε*2* PD mouse model^16^. The lack of evidence for the relationship between the *APOE* genotype and motor decline might be explained by the fact that a PD mouse model has differences in behavior, physiology, or pathology compared with PD patients^32^. For example, A53T-Tg mice exhibit progressive motor dysfunction, but overt degeneration of nigral dopamine neurons has not been observed in A53T-Tg mice^33,34^. Also, A53T mouse model relies on the overexpression of a mutant transgene, in which expression amounts are affected by interaction with other proteins, suggesting a different amount of alpha-synuclein in the human and mice brain^16^. On the other hand, the H&Y scale has only six options and therefore a large variety of impairment severities is collapsed together^35^. Therefore, it might be less sensitive to motor progression and influence the lack of evidence for the association between *APOE* and motor progression. Further longitudinal studies using the detailed motor scale of PD are required to see the effect of *APOE* genotype on motor progression.

The annual rate of decline in MMSE score was − 2.8 and − 1.8 points in the early fast decline and fast decline groups, respectively, which were significantly larger than the 0.0 points per year in the stable group. The DeNoPa study evaluated the annual decline in MMSE score and found that the MMSE scores declined by 0.2 points per year^4^, which was similar or slightly larger than the rate of 0.1 points per year found in our study. By using group-based trajectory modeling, we discovered interindividual variation in the annual rate of decline in MMSE score, which correlated well with prognosis in terms of conversion to PD with dementia. In addition, multivariate linear regression analysis for annual changes in MMSE scores revealed that older age at disease onset, presence of the *APOE* ε4 allele, a depressive mood at the baseline visit, and higher initial H&Y stage were associated with higher cognitive decline rates. Older age at onset was associated with cognitive decline in previous longitudinal studies^23,36^. Preceding depressive mood was associated with poor cognitive function in a previous longitudinal study of PD and also in a study of the general population^25,37^. Neural networks including the prefrontal cortex and hippocampus are functionally altered in a depressive state^38^. Several longitudinal studies revealed that severe baseline motor symptoms were associated with more rapid cognitive decline^23,36,39^. There might be common risk factors for high initial H&Y stage and fast cognitive decline, such as genetic factors other than *APOE*, which were not evaluated in this study. The predictors of progression of cognition in previous studies included cardiovascular risk factors, Rapid eye movement sleep behavior disorder (RBD), and serum inflammatory markers^4^. We did not find cardiovascular risk factors, heavy alcohol drinking, smoking, and RBD to be significant predictors for cognitive decline. Future large-scale cohorts are necessary to investigate the environmental risk factors as predictors for cognitive decline.

Interestingly, cognitive trajectories were associated with motor decline, although *APOE* ε4 allele was only associated with cognitive decline. Cross-sectional studies revealed that overall cognitive impairment correlated with motor impairment but not with disease duration^40,41^. Confounding factors, especially genetic factors such as *GBA*, which were not evaluated in this study might have contributed to the common pathophysiology of the simultaneous progression of motor and cognitive symptoms.

We are aware of some limitations to our approach. First, we chose MMSE scores to assess global cognition. MoCA test has been considered to be more sensitive in detecting mild cognitive impairment in PD^26^, and in detecting cognitive decline between baseline and after 3.5 years of follow-up than MMSE^42^. However, one longitudinal cohort showed that MMSE changed significantly in contrast to the MoCA, especially in patients with disease duration > 10 years^43^. As median disease duration at the latest follow-up in this study was 11 years, a change in MMSE might have been detected during this period. Also, both MMSE and MoCA resulted in excellent discrimination of PD patients with dementia from those without dementia^26^. Second, we could not regularly assess cognitive function in all participants, which might have contributed to selection bias. Because the rate of dementia in this study was similar or slightly lower than what has been reported in previous studies^4^, we thought that the effect of selection bias was not a critical issue. Third, we chose the H&Y stage instead of the Unified Parkinson’s Disease Rating Scale (UPDRS), which assesses motor symptoms in detail and is more sensitive to change. The H&Y scale is the most widely used scale to estimate the severity of PD, and it has shown a good correlation with UPDRS^28^. As mentioned above, this relationship needs validation in independent patient cohorts using detailed motor scale. Fourth, non-motor symptoms were collected via the interview, but not using quantitative scales. Also, we could not collect data for anxiety, apathy, and excessive daytime sleepiness, which are known to be associated with PD and cognition. Finally, the number of patients in the early fast decline and fast decline groups was small, and it might have led to the lack of differences in other risk factors. However, despite the small number of patients in the “early fast decline” and “fast decline” groups, age at disease onset, education, and *APOE* genotype showed significant differences between groups.

In conclusion, we identified cognitive trajectories of PD, which had a strong positive correlation with the prognosis. The presence of the *APOE* ε4 allele, combined with older age at onset, depressive mood, and higher initial H&Y stage, contributed to a faster cognitive decline. However, we did not find associations between either *APOE* ε4 or *APOE* ε2 with motor progression in PD. Therefore, *APOE* genotype could be used to predict the cognitive trajectory in PD.

## Methods

### Subjects

We retrospectively reviewed the PD patient registry of Asan Medical Center, which includes patients recruited from 2011 to 2016 for genomic analysis. The patient registry is described in a previous study^44,45^. A total of 1,050 patients with PD based on the UK Brain Bank criteria were included, regardless of disease duration or cognitive status. At study enrollment, patients underwent general cognitive assessment using the Korean version of the MMSE^46^, and were assessed for motor symptoms using the Hoehn and Yahr (H&Y) stage by a movement disorder specialist (SJC). For this study, we selected patients with PD without dementia at study enrollment, who underwent a subsequent MMSE at an interval of at least 5 years to assess the long-term change in global cognition.

**Figure 5:**
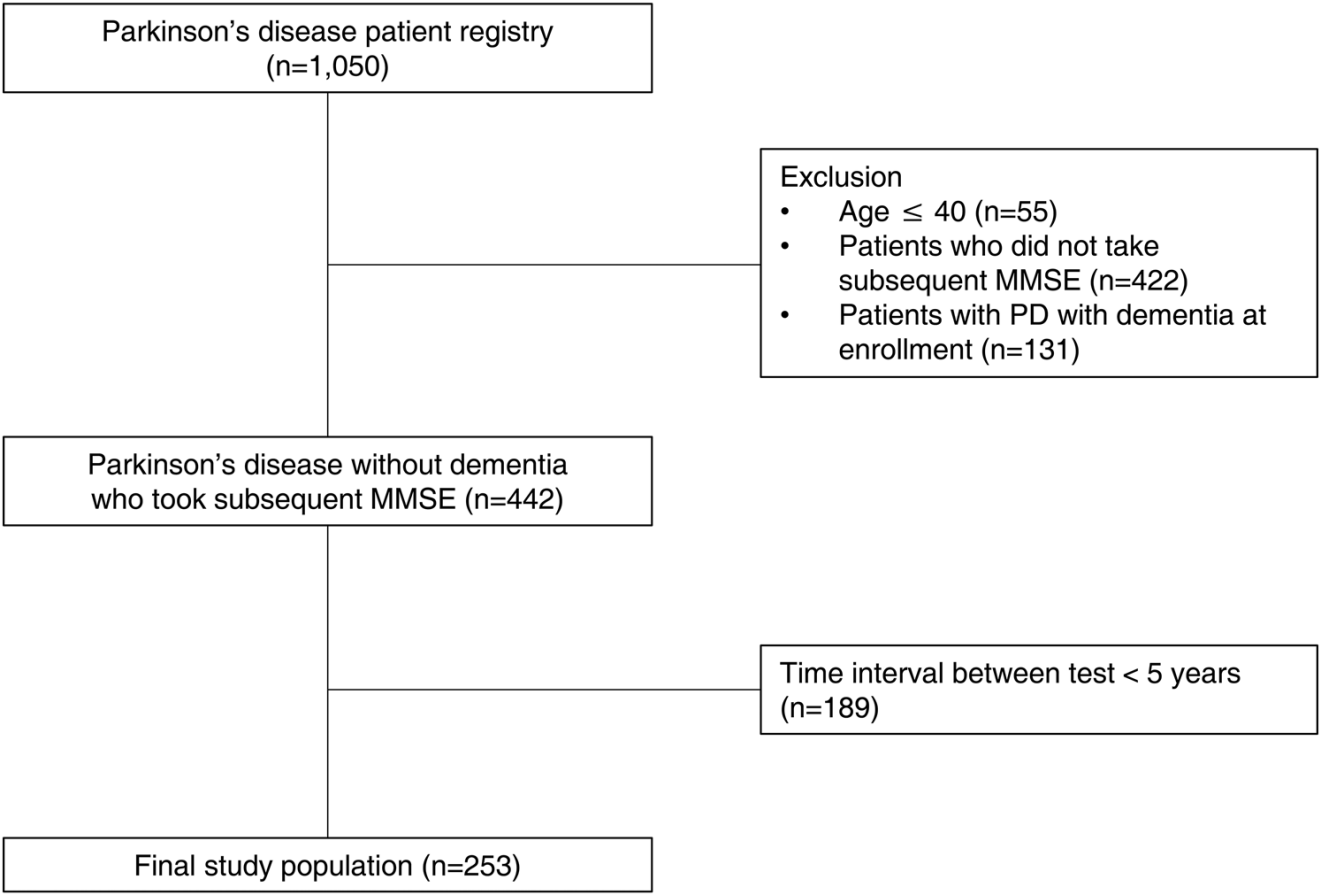
Study flowchart.

Among the 1,050 participants in the PD patient registry, patients aged less than 40 years at the time of diagnosis (n = 55) were excluded, because patients with young-onset Parkinson’s disease have different clinical features than late-onset PD. We excluded 422 patients who did not take a subsequent MMSE, and 131 patients having PD with dementia (PD-D) at enrollment, based on the diagnostic criteria proposed by the Movement Disorder Society Task Force^47,48^. To diagnose PD-D, patients with PD and/or their caregivers were asked questions about cognitive decline, and daily functioning such as the patient’s ability to manage finances, use pieces of equipment and cope in social situations at study enrollment. In cases with evidence of impairments in daily life due to cognitive change or definite cognitive decline on MMSE, detailed neuropsychological tests (e.g. Seoul Neuropsychological Screening Battery)^49^ were conducted to specify the pattern of cognitive deficits and to diagnose PD-D at level II in most patients. The diagnosis of PD-D was achieved by consensus between two neurologists. The final study population included 253 patients with PD, whose time interval between MMSE tests was over 5 years (Fig. 5). We confirmed that no patient met the clinical diagnostic criteria for dementia with Lewy bodies^50^. No patient had a family history suggesting autosomal dominant disease. All patients were unrelated and ethnic Koreans without any foreign ancestry. This study was approved by the Institutional Review Board (IRB) of Asan Medical Center and was performed in accordance with relevant named guidelines and regulations. All patients provided an informed consent form following IRB regulations.

### Assessment of cognitive decline

Patients with PD visited the outpatient clinic at 3- to 6-month intervals. On every visit, conversion to dementia was assessed. As a routine practice, patients with PD and/or their caregivers were asked questions about cognitive decline, and daily functioning, such as the patient’s ability to manage finances, use pieces of equipment, and cope in social situations, same as at enrollment. Patients were encouraged to undergo serial MMSE assessments at intervals of least 2–3 years. If significant cognitive changes and subsequent impairment on daily life activities or definite cognitive decline on MMSE were detected (level I), detailed neuropsychological tests (e.g. Seoul Neuropsychological Screening Battery)^49^ were conducted to specify the pattern of cognitive deficits and to diagnose PD-D at level II in most patients. The diagnosis of PD-D was made by two neurologists, based on the clinical diagnostic criteria proposed by the Movement Disorder Society Task Force^47^.

Sixty-two patients underwent MMSE at two timepoints, 89 underwent MMSE at three timepoints, 70 underwent MMSE at four timepoints, 28 underwent MMSE at five timepoints, and five patients underwent MMSE at six timepoints. Performance on all measures was transformed into age-adjusted and education-adjusted z-scores using previously published normative data^48^.

### Assessment of motor and non-motor symptoms

At 3- to 6- months intervals, H&Y scores were evaluated by a movement specialist (SJC) during medication “‘on’’ state. We obtained H&Y stages from the baseline visit to the time of the final MMSE assessment. At study enrollment, patients were interviewed to identify non-motor symptoms including depressive mood, pain, fatigue, psychosis, RBD, and autonomic dysfunction (including orthostatic hypotension, sexual dysfunction, gastrointestinal dysfunction, sialorrhea, and sweating)^51^ and environmental risk factors of PD. Environmental risk factors included heavy alcohol drinking, previous or current smoking, and exposure to pesticides. Heavy drinking was defined as consuming seven or more drinks in a single day or drinking more than five days per week^52^.

### APOE genotype

Using DNA extracted from peripheral blood, the *APOE* genotype of each patient was analyzed using the Korean Chip (K-CHIP), which contains *APOE* rs429358 and rs7412. K-CHIP was designed by the Center for Genome Science, Korea National Institute of Health, Korea (4845–301, 3000–3031) (www.cdc.go.kr). Genomic analyses were conducted as described in the previous study^44^. We identified *APOE* ε2, ε3, ε4 alleles based on the single nucleotide polymorphism of rs429358 and rs7412. As the number of patients with homozygous *APOE* ε4 was very small (n = 2), we evaluated ε2/ε4, ε3/ε4, ε4/ε4 as the same group (ε 4 carriers). Because ε4 carriers have increased risk for dementia,^53^ we compared ε2/ε3 (ε2 carriers) and ε3/ε3 to determine the protective effect of *APOE* ε2. There was no patient with the ε2/ε2 genotype.

### Group-based trajectory modeling

Group-based trajectory models are designed to identify clusters of individuals following similar progressions of outcome over time^12^. Using serial MMSE z-scores, we performed group-based trajectory modeling with the *proc traj* SAS macro^12^, which involves making various models to determine the number of cognitive trajectories according to their respective slopes. The BIC was used to select the number of trajectories that best fit the data. The subject was assigned to the group with the highest posterior probability.

### Statistical analysis

We compared demographics and clinical characteristics across each trajectory groups using one-way analysis of variance for continuous variables and chi-square tests for categorical variables. Post-hoc analyses were performed using the Tukey’s honestly significant difference post-hoc test if the data with continuous variables met the assumption of homogeneity of variance, and Dunnett’s post-hoc test if the data with continuous variables did not meet the assumption of homogeneity of variance. For post-hoc analysis of categorical variables, we used Bonferroni’s correction. We used Student’s *t*-test or Pearson’s correlation test to identify factors associated with mean annual changes in MMSE score. Potential risk factors included baseline demographics, comorbidities, *APOE* genotype, environmental risk factors, non-motor symptoms, and H&Y stage at initial assessment. We used multivariate analysis with stepwise selection and further performed all analyses using a backward selection procedure to confirm the final model.

Kaplan–Meier analysis was performed to assess the progression of motor symptoms from diagnosis to the onset of H&Y stage 3 and H&Y stage 4 according to the *APOE* genotype, as described in the previous studies^54–56^. Also, Kaplan–Meier analysis was performed to assess the progression to dementia according to the *APOE* genotype. All data analyses were performed using R (version 3.3.1), and SAS (version 9.4; SAS Institute; Cary, NC), with the significance level set at 0.05 (two tailed).

## Supplementary Information

Supplementary Information.

## Data Availability

All deidentified data that support the findings of this study are available upon reasonable request to the corresponding author from other researchers if ethical approval is granted.
