# Supplementary material for: The role of APOE in cognitive trajectories and motor decline in Parkinson’s disease
Source: Sci Rep. 2021 Apr 9;11:7819. doi: 10.1038/s41598-021-86483-w (PMC8035327; doi:10.1038/s41598-021-86483-w)
Supplement: Supplementary file 1 — Supplementary Information. [file 41598_2021_86483_MOESM1_ESM.docx]

The role of *APOE* in cognitive trajectories and motor decline in Parkinson’s disease

Sungyang Jo^1^, Seon-Ok Kim^2^, Kye Won Park^1^, Seung Hyun Lee^1^, Yun Su Hwang^1^, Sun Ju Chung^1*^

^1^Department of Neurology, Asan Medical Center, University of Ulsan College of Medicine, Seoul, Republic ofKorea

^2^Department of Clinical Epidemiology and Biostatistics, Asan Medical Center, University of Ulsan College of Medicine, Seoul, Republic of Korea

**[Supplementary data]**

**Supplementary table S1. Number of patients in each H&Y stage**

|  | Stage 1 | Stage 2 | Stage 2.5 | Stage 3 | Stage 4 | Stage 5 |
| --- | --- | --- | --- | --- | --- | --- |
| Baseline HY Stage 1 | Baseline (n=28)→ | Progression (n=27) → | Progression (n=24) →  Missing=2 | Progression (n=19) → | Progression (n=3) → | (n=0) |
| Baseline HY Stage 2 |  | Baseline  (n=153) → | Progression (n=131) →  Missing=15 | Progression  (n=123) → | Progression (n=46) → | Progression  (n=6) |
| Baseline HY Stage 2.5 |  |  | Baseline  (n=28) → | Progression  (n=27) → | Progression  (n=17) → | Progression  (n=5) |
| Baseline HY Stage 3 |  |  |  | Baseline  (n=39) → | Progression  (n=27) → | Progression  (n=5) |
| Baseline HY Stage 4 |  |  |  |  | Baseline  (n=5) → | Progression  (n=2) |

Abbreviations: H&Y = Hoehn and Yahr

**Supplementary Table S2. Clinical manifestations including cognitive decline at the final assessment**

|  | Early fast decline (n =6) | Fast decline  (n = 16) | Gradual decline (n = 62) | Stable  (n = 169) | *p* |
| --- | --- | --- | --- | --- | --- |
| Follow-up  duration (y) | 5.0 (5.0−5.0) ^a^ | 7.0 (6.0− 8.0) | 7.5 (6.0− 8.0) | 8.0 (6.0− 9.0) | 0.007 |
| Conversion to dementia | 6 (100.0) | 12 (75.0) | 34 (54.8) ^d^ | 28 (16.6) ^a^ | < 0.001 |
| Years from PD diagnosis to dementia | 4.0 (1.0−6.0)^b^ | 7.0 (6.0−7.5)^c^ | 8.5 (6.0−12.5) | 10.0(7.5−16.0) | 0.005 |
| Age at final assessment (y) | 71.0 (67.0−80.0) | 74.0 (66.0−79.0) | 74.0 (70.0−78.0) ^c^ | 69.0 (63.0−75.0) | 0.001 |
| Final MMSE (z) | -10.5  (-15.4−-8.1) | -7.7  (-9.1−-6.7) | -2.9  (-3.7−-2.0) ^d^ | -0.3  (-1.1− 0.3) ^a^ | < 0.001 |
| Final MMSE (raw) | 12.0 (10.0−17.0) ^a^ | 16.5 (11.0−19.5) | 22.5 (19.0−24.0) ^d^ | 27.0 (25.0−29.0) ^a^ | < 0.001 |
| Annual change in MMSE (z) | -1.8  (-2.3−-1.3) ^a^ | -1.1  (-1.4−-0.8) ^a^ | -0.3  (-0.5−-0.2) ^d^ | -0.0  (-0.1− 0.1) ^a^ | < 0.001 |
| Annual change in MMSE (raw) | -2.8  (-3.0−-2.2) ^a^ | -1.8  (-2.2−-1.4) ^a^ | -0.6  (-1.2−-0.4) ^d^ | -0.1  (-0.3− 0.0) ^a^ | < 0.001 |

Abbreviations: MMSE = Mini-Mental State Exam; PD = Parkinson’s disease.

^a^Significant difference compared with all the rest of the groups, using Dunnett’s post-hoc test

^b^Significant difference compared with the gradual decline and stable group, using Dunnett’s post-hoc test

^c^Significant difference compared with the stable group, using Dunnett’s post-hoc test

^d^Significant difference compared with the early fast decline and fast decline groups, using Dunnett’s post-hoc test

**Supplementary Table S3. Univariate linear regression showing factors significantly associated with the mean annual change in MMSE in patients with Parkinson’s disease**

|  | B | SE | *p* | |
| --- | --- | --- | --- | --- |
| Age at disease onset | -0.028 | 0.005 | < 0.001 | |
| Female | 0.089 | 0.099 | 0.37 | |
| Education (y) | 0.009 | 0.010 | 0.36 | |
| Disease duration at enrollment (y) | 0.010 | 0.013 | 0.44 | |
| Initial MMSE (raw) | -0.001 | 0.019 | 0.98 | |
| Vascular risk factor at study enrollment (%) | | | | |
| Hypertension | -0.170 | 0.114 | 0.14 | |
| Diabetes mellitus | -0.175 | 0.160 | 0.28 | |
| Hyperlipidemia | 0.154 | 0.144 | 0.29 | |
| Cardiac disease | -0.032 | 0.203 | 0.87 | |
| *APOE* genotype (%) |  |  |  | |
| ε4 carrier | -0.487 | 0.114 | < 0.001 | |
| ε2 carrier | 0.065 | 0.163 | 0.69 | |
| Environmental risk factor, n (%) | | | |  |
| Heavy alcohol abuse | 0.017 | 0.029 | 0.54 | |
| Smoking | 0.006 | 0.071 | 0.93 | |
| Use of pesticide | 0.088 | 0.093 | 0.34 | |
| Nonmotor symptom, n (%) | | | | |
| Depressive mood | -0.261 | 0.098 | 0.008 | |
| REM sleep behavior disorder | -0.002 | 0.103 | 0.98 | |
| H&Y stage | -0.238 | 0.079 | 0.003 | |

Abbreviations; H&Y = Hoehn and Yahr; MMSE = Mini-Mental State Exam;

**Supplementary table S4. Cox proportional hazards model showing factors significantly associated with motor progression from diagnosis to the onset of H&Y 3 in patients with Parkinson’s disease**

|  | Coefficient | Hazard ratio | 95% CI | *p* |
| --- | --- | --- | --- | --- |
| Age at disease onset | 0.001 | 1.00 | 1.00−1.02 | 0.972 |
| Disease duration at enrollment (y) | -0.250 | 0.78 | 0.74−0.82 | < 0.001 |
| Trajectory group^a^ |  |  |  |  |
| Early fast decline | 2.110 | 8.25 | 3.45−19.75 | < 0.001 |
| Fast decline | 1.087 | 2.97 | 1.73−5.10 | < 0.001 |
| Gradual decline | 0.554 | 1.74 | 1.26−2.40 | < 0.001 |

Abbreviations; H&Y = Hoehn and Yahr

^a^ Reference: stable groups
